# Bilateral myositis ossificans of the masseter muscle after chemoradiotherapy and critical illness neuropathy- report of a rare entity and review of literature

**DOI:** 10.1186/1758-3284-1-30

**Published:** 2009-08-12

**Authors:** Astrid L Kruse, Christine Dannemann, Klaus W Grätz

**Affiliations:** 1 University Hospital Zurich, Department of Craniomaxillofacial and Oral Surgery, Frauenklinikstr, 24 CH-8091 Zurich, Switzerland

## Abstract

Myositis ossificans in the head and neck is a rare heterotropic bone formation within a muscle. Besides fibrodysplasia ossificans progressiva, traumatic and neurogenic forms are described in the literature.

We are presenting the case of a 35-year-old female patient with a very rare form of MO of both masseter muscles after 4 weeks of intensive care because of complications (critical illness neuropathy) after chemotherapy.

Therefore, special attention should be paid to surgical trauma. As in the present case, radiotherapy, long-time intubation with immobilization and critical myopathy and neuropathy can cause MO with severe problems, such as trismus and reduced mouth hygiene, which can lead to reduced quality of life.

## Introduction

Myositis ossificans is a heterotropic bone formation within a muscle. The incidence in head and neck is rare; it is mainly found (80%) in the extremities [1]. Concerning the head and neck, involvement of the temporal muscle [2], the masseter muscle [3], the buccinators muscle [1], the platysma muscle [4] and the sternocleidomastoid muscle [5] is described. Myositis ossificans can be divided into subtypes (Table 1). The fibrodysplasia ossificans progressiva, a rare autosomal-dominant disorder, has a prevalence of 1 in 2 million people and an onset in early childhood [6].

**Table 1:** Clinical subdivision of MO

*Fibrodysplasia ossficans progressiva*	Symmetric congenital malformations of great toes, ossification of upper extremities and back
*Traumatic MO*	Circumscript new bone growth within the muscle after injury

*Neurogenic MO*	Occurs below spinal-cord injury

Traumatic myositis ossificans, also called Myositis ossificans circumscripta, is the most common form, resulting in an ossification of muscle after trauma or inflammation.

### Case

A 35-year-old female patient was referred because of the progressive decrease in the range of motion of her mandible for 2 years. Three years before, she underwent chemoradiotherapy because of an Epstein-Barr virus associated nasopharyngeal carcinoma (cT4cN2bMo). After the 2cd chemotherapy with platinol/5-FU, she developed an aplasia with 0.03 *10^3^/μl leucocytes (initially 4.5). In the course of the disease she developed several complications, such as pseudomonas sepsis, renal insufficiency, hepatopathology and repeating asystole caused by cardio toxic effect of 5-Fluorouracil. Finally she revealed a critical illness neuropathy while being intubated for 4 weeks in an intensive care unit.

**Figure 1:**
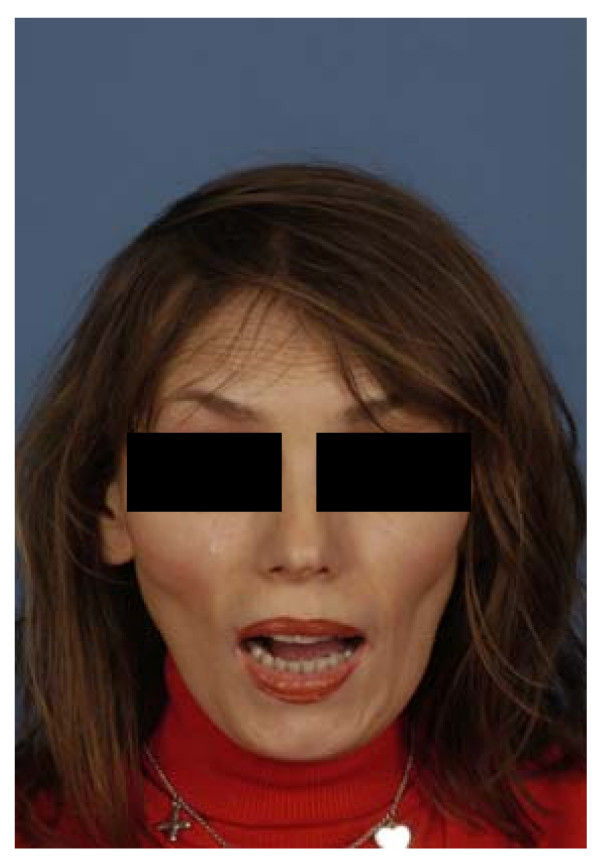
**Patient with max. mouth opening**.

Clinical examination revealed a strong tenderness of both masseter muscles, anterior to the superior border of the ramus and reaching from the zygomatic arch to the body of the mandible without pain on palpation. Additionally, no pain was elicited on palpation of the temporomandibular joint on both sides. The maximum incisal opening was 1 cm with limited lateral excursions (Fig. 1). Limited intraoral examination revealed reduced mouth hygiene, several teeth with severe dental caries, and a bony hard mass in both buccal sulci.

**Figure 2:**
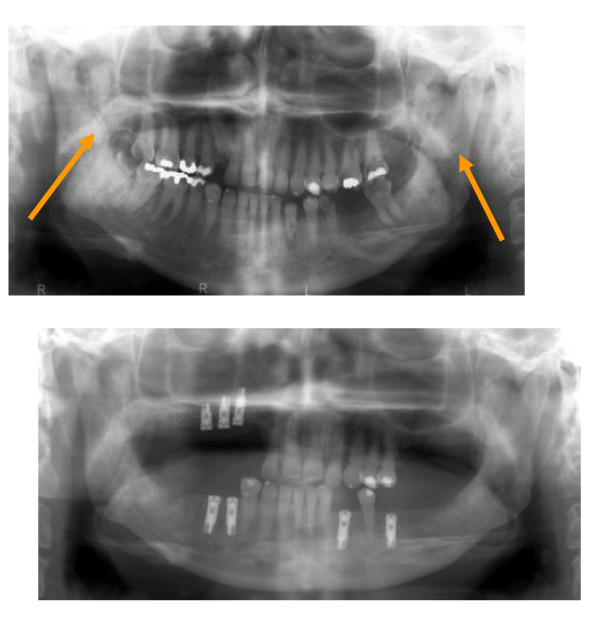
**a. Panoramic radiograph with radiopaque mass (arrow)**. b. After insertion of implants

The panoramic radiograph revealed an amorphous calcification within the soft tissue on both sides and multiple dental caries (Fig. 2a and Fig. 2b).

**Figure 3:**
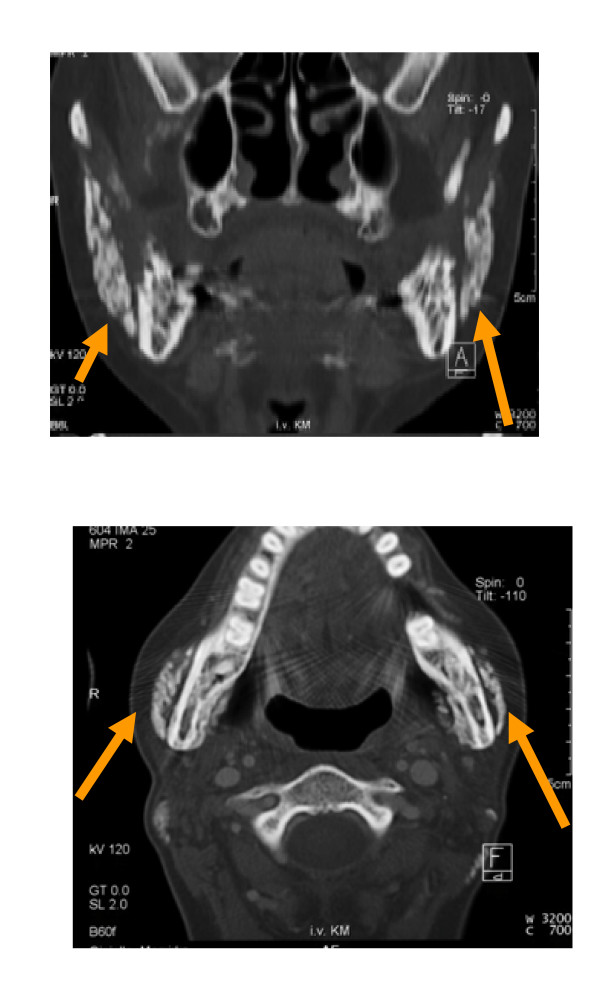
**a and b**. Calcified mass (arrow) in the masseter muscle on both sides.

Axial and coronal computed tomography scans were obtained of the facial skeleton, and bilateral diffuse calcification of the masseter muscles without involvement of temporal and pterygoid muscles or temporomandibular joint on both sides was seen (Fig. 3a and Fig. 3b). A well-circumscribed calcified mass with density similar to bone was clearly separated from the adjacent mandibular ramus. Laboratory data were all within normal limits.

The history of long immobilisation, in combination with critical illness neuromyopathy, radiotherapy, clinical examination, and radiographic findings on both masseter muscles, was strongly suggestive of the diagnosis of myositis ossificans. Extraction of caries teeth and an active mouth opening were performed using anaesthetic intubation (fiberoptic nasal intubation). As a second step, 8 months later, 3 implants were inserted in the upper jaw and 4 implants in the lower jaw (Fig. 2b). Concerning trismus, physical therapy and TheraBite^® ^training have been initiated, but the mouth opening is still unchanged (1 cm). The patient is still seen by us once a year for control and regularly by a dental hygienist.

## Discussion

The pathophysiology is still unclear. Bone morphogenetic protein expression is discussed because of muscle trauma resulting in stem cells differentiating into osteoblasts and finally leading to ossification. Therefore, one treatment strategy can be bone morphogenic protein type I receptor inhibition in order to reduce this heterotropic ossification [7]. Aho et al. [8] were able to show in a rabbit model that microinjury and subsequent muscle necrosis can cause invasion of macrophages and release osteogenic growth factors.

Concerning the aetiology of myositis ossificans traumatica of the masticatory muscles, several causes have been described: tooth extraction associated with involvement of the buccinator muscle [1], local anaesthesia with involvement of the pterygoid muscle, and direct force with myositis ossificans traumatica of the masseter. In table 2 all cases with masseter involvement between 1954 and 2008 is presented.

**Table 2:** overview of masseter involvement between 1954 and 2008

**Author**	**Year**	**Masseter Side**	**Aetiology**
Yano [15]	2005	bilateral	Struck on both cheeks

Aoki [16]	2002	one	Struck on the cheek

Geist [17]	1998	one	Mandible fracture

Myoken [18]	1998	one	Trauma

Steiner [3]	1997	One	Mandible fracture
		one	Shotgun

Lello [19]	1986	one	Struck on the cheek

Arima [20]	1984	one	Struck on the cheek

Christmas [21]	1982	one	Fall from horse

Beck [12]	1954	bilateral	Lower jaw fracture

Different facts are important for the development of myositis ossificans on both masseter muscles in the present case. On the one hand, the neurogenic component plays an important role because of long time intubation and immobilization with incomplete pareses C3 and critical myopathy/neuropathy (CIPNM) in an intensive care unit. On the other hand, the traumatic/inflammatory component plays an important role with sepsis. CIPNM occurs in 25–63% of patients who have been on an artificial respirator for at least 1 week [9]. In patients with sepsis, the incidence increases, and male patients develop this condition about twice as often as females [9].

Microcirculatory damage can reduce delivery of oxygen and glucose and therefore affect the nervous system, leading to CIPNM with selective myosin loss in muscles fibres and myonecrosis by inflammatory factors [10]. Factors that are associated with the development of CIPNM can be physical, surgical trauma, or chemicals in combination with sepsis and the application of neuromuscular blocking agents and steroids. These neuromuscular blocking agents are metabolized by the liver and kidney [11]. If these organs fail, as in the present case, the effect of these blocking agents will be prolonged. Flaccid weakness of neck flexors and facial muscles are described as a feature in CIPNM [11], but cranial nerve involvement seems to be rare [9]. But no case in combination with myositis ossificans has been described in the literature.

Another important factor could be the tube change with forced mouth opening without regular oral physiotherapy while being intubated without leading to a bleeding in an already fibrotic muscle after previous radiotherapy.

Bilateral involvement of the masseter muscles is extremely rare. The first case in the literature was presented in 1954 by Beck [12].

Different treatment strategies have been discussed in the literature for myositis ossificans traumatica: surgical treatment (excision), physiotherapy (including TheraBite), medical therapy (non-steroidal anti-inflammatory drugs, bisphosphonates and magnesium), and low-dose radiation therapy [13]. Low-dose radiation therapy and non-steroidal anti-inflammatory drugs are used in order to inhibit mesenchymal differentiation into osteoblasts. The use of bisphosphonates in order to prevent myositis ossificans is described in orthopaedics [14].

An excision was not an option in the present case because of the complete involvement of both masseter muscles. Medical therapy was not offered because the patient was presented with a 2-year history of complaints, so the early phase of calcification was already over. The use of etidronate can also lead to osteomalzia with bisphosphonate-associated necrosis of the jaws, so the patient did not receive this agent.

## Summary

Myositis ossificans is a rare complication that can be caused by muscle trauma. Therefore, special attention should be paid to surgical trauma. As in the present case, radiotherapy, long-time intubation with immobilization and critical myopathy and neuropathy can cause myositis ossificans with severe problems, such as trismus and reduced mouth hygiene, which can lead to reduced quality of life. Hence, in cases involving trismus, after a long period of immobilisation, early intensive physiotherapy is indicated.

## Competing interests

The authors declare that they have no competing interests.

## Consent

Written informed consent was obtained from the patient for publication of this case report and accompanying images. A copy of the written consent is available for review by the Editor-in-Chief of this journal.

## Authors' contributions

AK carried out the analysis of the patient's data and literature review, CD also participated in the analysis of the patient's data and literature review and KWG participated in the design of the study and coordination. All authors have read and approved the final manuscript.
